# Merrell, Thorp & Loyd

**Published:** 1877-07-20

**Authors:** 


MERRELL, THORP & LOYD would respectfully solicit a careful perusal of their circular, appearing as an insert in the present number of THE RECORD. It is a full explanation of specific tinctures and fluid extracts, and is of interest to the profession. It is the circular of a house making the highest standard of medicines attainable, and claims always unfailing uniformity.

